# Histological and morphometric changes in cardiac conduction fibers after spontaneous myocardial infarction in horses and dogs

**DOI:** 10.14202/vetworld.2025.827-836

**Published:** 2025-04-19

**Authors:** Fabián Gómez-Torres, Luis Ballesteros-Acuña, Amparo Ruíz-Sauri

**Affiliations:** 1 Department of Basic Sciences, School of Medicine, Universidad Industrial de Santander, Cra 32 # 29-31, 68002, Bucaramanga, Colombia; 2 Department of Pathology, Faculty of Medicine and Odontology, University of Valencia, Av. de Blasco Ibáñez, 15. 46010. Valencia, Spain; 3 INCLIVA Biomedical Research Institute, Av. de Blasco Ibáñez, 17. 46010, Valencia, Spain

**Keywords:** cardiac arrhythmias, cardiac conduction fibers, conduction cells, dog, horse, myocardial infarction

## Abstract

**Background and Aim:**

Arrhythmic sudden cardiac death in dogs and horses often results from ventricular arrhythmia secondary to myocardial damage. Despite this, limited data exist on the histomorphometric changes in cardiac conduction fibers (CCFs) and cardiac conduction cells (CCCs) following spontaneous myocardial infarction (MI). This study aimed to characterize morphometric and histological alterations in conduction fibers and their junctions with cardiomyocytes in infarcted hearts of horses and dogs.

**Materials and Methods:**

Ten hearts from horses and 10 from dogs that had died suddenly were examined. Histological and immunohistochemical analyses were performed using hematoxylin and eosin, Masson’s trichrome, and periodic acid–Schiff staining to identify conduction fibers and assess glycogen accumulation. The thickness and density of conduction fibers, as well as the diameter of conduction cells, were measured using image analysis software. Statistical comparisons were conducted using t-tests, analysis of variance, and Cohen’s d-test.

**Results:**

In horses, the diameter of CCCs was significantly smaller in infarcted cases (55.74 μm) compared to normal hearts (79.08 μm) (p < 0.001). In dogs, slight hypertrophy of CCCs (31.21 μm) was observed in normal hearts, whereas infarcted hearts exhibited reduced diameters (26.83 μm) (p = 0.114). The density of CCFs was 9.06% in horses and 7.99% in dogs (p = 0.846), while fiber thickness was 30.06 μm in horses and 29.86 μm in dogs (p = 0.263). Horses exhibited extensive myocardial fibrosis, particularly in the middle third and posterior left ventricle, while dogs displayed milder lesions distributed across the ventricle.

**Conclusion:**

This study demonstrates a reduction in CCC size in horses and minor hypertrophy in dogs, coupled with fibrotic myocardial lesions of varying severity. The observed histomorphometric changes provide insight into the structural impact of MI on conduction cells, which may contribute to ventricular arrhythmias in these species. These findings have implications for veterinary cardiology and the management of MI-related arrhythmic conditions.

## INTRODUCTION

The histological structure of the ventricular cardiac conduction system is composed of fibers and conduction cells. Cardiac conduction fibers (CCFs) are formed by cardiac conduction cells (CCC) and the small connective tissue sheaths surrounding them [1]. Arrhythmic sudden death (SD) in dogs is a result of ventricular arrhythmia secondary to pathological myocardial damage, such as dilated cardiomyopathy, arrhythmogenic right ventricular cardiomyopathy, myocardial hypertrophy, and myocarditis [2–5]. Moreover, it has been related to ventricular fibrillation, which is the most probable mechanism of canine SD, although little has been documented [5–8]. Ventricular arrhythmias following myocardial infarction (MI) are thought to be due to an alteration in the electrophysiological properties of cardiac fibers in the infarct scar [9–12]. Cardiac conditions in racehorses are often suspected of causing SD or collapse [13–15]. SD in racehorses is often related to exercise and has been associated with axial skeletal fracture, exercise-induced pulmonary hemorrhage, vessel rupture and internal bleeding, myocarditis or other cardiovascular diseases, and injection-associated injuries [13, 16, 17]. In dogs, in which infarction was induced by occlusion of the coronary arteries; evident coagulation necrosis was observed in the cardiomyocytes; and eosinophilia was observed, loss of striae, and disappearance of nuclei in these cells.

The interstitium was infiltrated by neutrophils, macrophages, and lymphocytes. In all preparations, the most superficial CCFs were normal and did not show changes characteristic of infarction or necrosis [9, 18]. Similar changes have also been observed in horses that have died of SD from different causes in various countries [17]. Thoroughbred horses succumbing to SD presented focal or multifocal areas of fibrosis or myocarditis between 1.5 and 2.1 cm in diameter in the interventricular septum and/or the free wall of the left ventricle (LV) [19]. Similarly, fibrotic lesions in the myocardium have been described and classified into mild, moderate, and severe grades. In this classification, mild lesions are very small and are characterized by a dense proliferation of collagen fibers and edema in the intramyocardial tissue. Moderate lesions were slightly larger and characterized by relatively dense fibrous tissue with loss of muscle fibers, whereas severe lesions had extensive loss of muscle fibers, which were replaced by dense collagen fibers and adipose tissue [16]. In a study of cardiac lesions with clinical evidence of cardiac disease in older horses (>10 years) in a slaughterhouse, approximately 94% of the horses had myocardial fibrosis and fatty infiltration of the myocardial fibers [20]. In a study of an Arabian horse, CCFs in the interventricular septum and right ventricular free walls were surrounded by a large amount of fibrous connective tissue near areas of myocardial atrophy. In interstitial fibrotic areas, perivascular infiltrates of lymphocytes and macrophages are minimal [21]. In the context of fibrosis, vacuolated CCCs are found in dogs, with slightly contracted myofibrils and some showing glycogen depletion or signs of intercalated disc dissociation [18]. In horses, subendocardial CCCs of the LV were decreased in size with mild variable interstitial fibrosis in SD cases [19].

Despite extensive research on MI and its consequences in humans, the specific histomorphometric alterations in the cardiac conduction system of horses and dogs following spontaneous MI remain poorly characterized. Previous studies have largely focused on clinical manifestations, ventricular arrhythmias, and gross pathological changes in these species, but few have provided detailed insights into the structural modifications of CCFs and CCCs. The extent to which MI alters the density, thickness, and connectivity of conduction fibers remains unclear, particularly in relation to their potential role in arrhythmogenesis. Moreover, while histological and immunohistochemical methods have been employed in normal cardiac tissue, their application to infarcted conduction fibers in veterinary medicine has been limited. Understanding these structural changes is essential for elucidating the mechanisms underlying conduction disturbances and sudden cardiac death in affected animals.

This study aims to describe the morphometric and histological alterations in CCFs and conduction cells in different regions of the LV and their junctions with cardiomyocytes following spontaneous MI in horses and dogs. By employing histological and immunohistochemical techniques, this research seeks to quantify changes in fiber thickness, density, and cellular morphology while also assessing myocardial fibrosis and conduction of fiber-myocardial junctions. The findings will provide critical insights into the structural integrity of the conduction system post-MI and its potential role in the development of arrhythmias in these species.

## MATERIALS AND METHODS

### Ethical approval

The procedures were in accordance with the Ethics Committee of the Universidad Industrial de Santander (N° 013–2023) and conform to Law 84 of 1989 of national scope, corresponding to Chapter VI of the “National Statute for the Protection of Animals,” on the use of animals in experiments and research.

### Study period and location

This study was conducted from November 2021 to November 2023 in the city of Bucaramanga, Colombia.

### Sample collection and processing

We used 10 hearts of horses (weighing 250–300 kg and 6–8 years old; heart weight 1036 ± 403.8 g) and 10 hearts of dogs (medium breed adult dogs weighing 8–19 kg and aged 10–14 years; heart weight 84.4 ± 37.7 g) with SD (understood as the death of the individual without any previous manifest morbidity) from necropsies performed by clinical veterinarians and in the city of Bucaramanga, Colombia. These hearts were evaluated morphologically and those found to have defined hyperemic areas in the ventricular walls and concomitant vascular obstruction of the corresponding artery was sampled for histopathological analysis. The patients with MI were included in this study.

Hearts were collected and fixed in a 5% formaldehyde solution. The LVs were divided into three slices of equal thickness (base, middle third, and apex), each of which was further divided into four regions (anterior, lateral, posterior, and septal) for histopathological analysis. Samples were embedded in paraffin and labeled for identification. In all regions, 5-mm thick samples were cut at the CCF site. Subsequently, each section was stained with hematoxylin and eosin and Masson’s trichrome.

In addition, we visualized the glycogen accumulated in the cytoplasm of CCCs and verified their identification using a periodic acid-Schiff (PAS) method (Cytek Biosciences, USA). Similarly, immunohistochemical staining was performed with the Anti-Human Desmin clone D33-IR606 (Dako Corporation, Agilent, USA) at 1/90 to visualize and improve the identification of the intermediate myofilaments (desmin) in these cells. The data from our research are comparable with previous study by Asakawa *et al*. [21] conducted on normal hearts using the same methodology.

### Morphometric and histological analysis

A total of 1300 histological images between the two species (900 in horses and 400 in dogs) were evaluated for the CCF study using a Leica DMD108 optical microscope (Leica Microsystems, Wetzlar, Germany). The computerized morphometric study was conducted using Image-Pro Plus 7.1 software (Media Cybernetics, Silver Spring, MD, USA). The microscope used was calibrated in the morphometry program using micrographs on a scale. Each analyzed micrograph was then calibrated within the measurement program, and the different measurements were taken using automatic segmentation for the total area and manual segmentation for the CCF area. Two researchers performed manual segmentation. Each micrograph was calibrated and the area of CCC and tissue was determined in μm^2^ by 10× segmentation. CCF density was calculated as the area occupied by the fibers compared to the total area of tissue. In addition, we measured the thickness of the CCF bundles at 10× transversally. In CCCs and cardiomyocytes, the area, maximum diameter, minimum diameter, mean diameter, and roundness were measured at 20× magnification. In each micrograph, we recorded the presence or absence of junctions between CCCs and cardiomyocytes called conduction fiber-myocardial junctions (CFMJs), checking for three previously described types: Contact through cell bodies (CCBs), contact through cell prolongations (CCPs), and contact through transitional cardiomyocytes (CTCs) [22, 23]. The presence of fibrotic lesions in MI was evaluated according to the criteria of Kiryu *et al*. [16]. Finally, the parameters were compared between the species.

### Statistical analysis

All categorical variables are expressed as percentages, while continuous variables are presented as mean ± standard deviation (SD). Statistical significance was set at p < 0.05. Data analysis was conducted using the Statistical Package for the Social Sciences (SPSS) 20 (SPSS, Chicago, IL, USA) and Microsoft Excel 2013 (Microsoft, Washington, USA).

The Chi-square test was employed to compare categorical variables, particularly for evaluating the presence and type of CFMJs. The Kolmogorov-Smirnov test was performed to assess the normality of morphometric parameters. For continuous variables, statistical tests were chosen based on normality and homoscedasticity assumptions. One-way analysis of variance was used for comparisons of normally distributed continuous variables across multiple groups, and Bonferroni correction was applied for *post hoc* analysis to account for multiple comparisons. If the normality assumption was violated, the Kruskal–Wallis test was used as a non-parametric alternative.

For pairwise comparisons, Student’s t-test (two-tailed) was applied for normally distributed morphometric parameters between horses and dogs, while the Mann–Whitney U test was used in cases of non-normal distribution. Cohen’s d-test was used to quantify the magnitude of differences in Student’s t-test comparisons, categorizing effect sizes as tiny (d < 0.2), small (0.2–0.5), moderate (0.5–0.8), large (0.8–1.2), or very large (d > 1.2) (Table 1).

**Table 1:** Meaning of Cohen’s d-test value (d).

d-value	Meaning
<0.1–0.2	Tiny
0.2–0.5	Little
0.5–0.8	Medium
0.08–1.2	Big
>1.2	Very big

This statistical framework ensured robust and reliable comparisons, allowing for a precise interpretation of morphometric and histological differences in conduction system structures between infarcted and non-infarcted hearts.

## RESULTS

### CCFs

#### Horses

**Figure 1:**
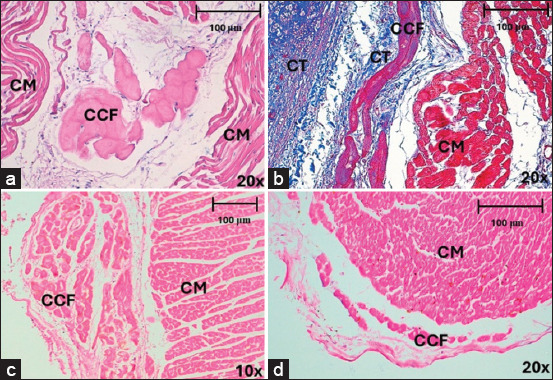
Cardiac conduction fibers of horses and dogs in different locations and regions, stained with Hematoxylin-eosin and Masson’s trichrome after myocardial infarction. (a) Cardiac conduction fibers in horses at the intramyocardial level, in the lateral region at 20× and (b) at the subendocardial level in the anterior region at 20×. (c and d) Cardiac conduction fibers in dogs at the subendocardial level, in the septal region at 10× and 20×. CCF=Cardiac conduction fibers, CM=Cardiomyocytes, CT=Connective tissue.

**Figure 2:**
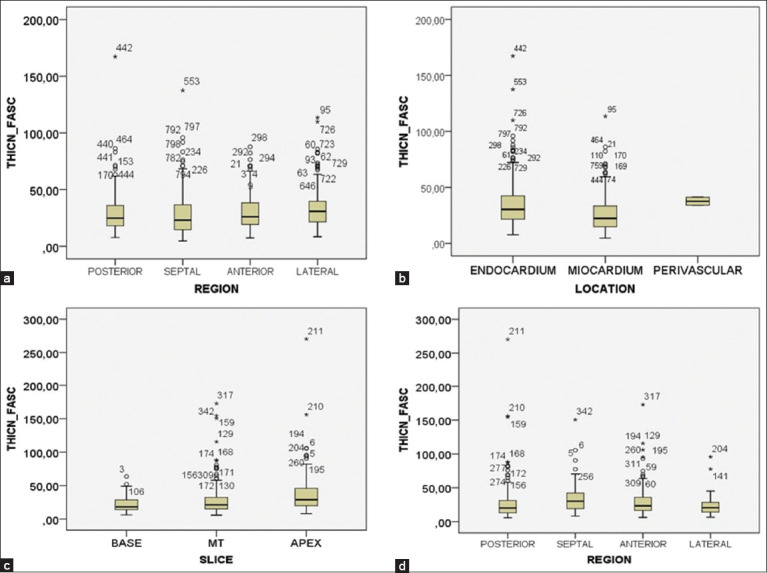
Statistical distribution of cardiac conduction fiber thickness by slice, region, or location in horses and dogs. Cardiac conduction fiber thickness by (a) region and (b) location in horses. Cardiac conduction fiber thickness by (c) slice and (d) region in dogs.

CCFs in horses were clearly identified in the subendocardium and myocardium by staining with hematoxylin-eosin (Figure 1a) and Masson’s trichrome (Figure 1b), showing higher density in the anterior region than in the septal (p = 0.017; df = 3914) or lateral regions (p = 0.003; df = 3,914). There were no significant differences in fiber density between slices (p = 0.072; df = 2,915) or locations (p = 0.668; df = 2,915) (Table 2). The CCF was statistically thicker in the lateral region than in the septal region (p < 0.001; df = 3,914) (Figure 2a). Likewise, the aforementioned values were higher in the endocardium than in the myocardium (p < 0.001; df = 2,915) (Figure 2b). There were no significant differences in CCF thickness among the slices (p = 0.072; df = 2,915).

**Table 2:** Mean density and thickness values of cardiac conduction fibers after MI according to section, region, and location in horses and dogs.

Slice and region	Horse		Dog	
	
	Conduction fiber density (%)	Fascicle thickness (μm)	Conduction fiber density (%)	Fascicle thickness (μm)
Slice				
Base	7.28	31.79	8.51	21.57
Middle third	10.29	28.99	7.19	28.93
Apex	8.62	29.07	8.01	39.83
Region				
Posterior	8.26	28.99	7.18	30.11
Septal	8.14	27.18	10.93	35.10
Anterior	12.66	31.19	8.55	31.11
Lateral	7.21	32.88	5.31	23.12
Location				
Subendocardium	9.38	34.27	7.74	30.14
Myocardium	8.10	25.43	-	-
Perivascular	12.22	37,60	8.46	15.23

MI=Myocardial infarction

#### Dogs

In dogs, CCFs were located in the subendocardium, and their cytoplasm had a similar coloration to that of cardiomyocytes, which complicated their differentiation (Figures 1c and d). The CCF density was higher in the septal region than in the posterior (p = 0.032; df = 3,363) or lateral regions (p = 0.001; df = 3,363). There was also a higher density of fibers in the anterior region than in the lateral (p = 0.050; df = 3,363), but no significant differences were found in density between slices (p = 0.441; df = 2,364). The CCFs were thicker at the apex than at the base (p < 0.001; df = 2,364) or middle third (p = 0.003; df = 2,364) (Figure 2c) and were thicker in the septal than in the lateral region (p = 0.050; df = 3,363) (Figure 2d). We found these fibers to be significantly thicker in the endocardium than at the perivascular level (p < 0.001; df = 2,364). The density of CCFs was 9.06% in horses and 7.99% (p = 0.846; d = 0.11) in dogs. The thickness of these fibers was 30.06 μm in horses and 29.86 μm in dogs (p = 0.263; d = 0.008) (Table 2).

### CCCs and cardiomyocytes

#### Horses

**Figure 3:**
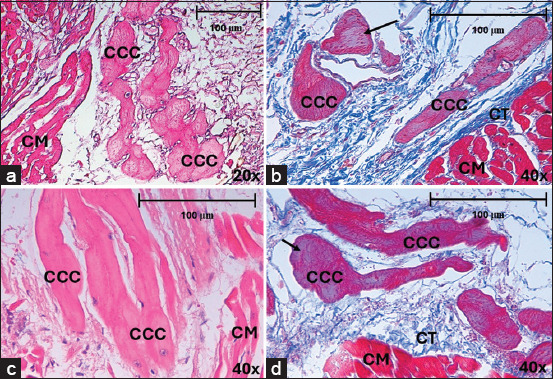
Morphology of CCCs in horses, stained with hematoxylin-eosin and Masson’s trichrome after myocardial infarction (a) CCCs in subendocardium, in the septal region at 20× and (b) the posterior region at 40×. (c) CCCs in myocardium, in the lateral region. (d) septal region at 40×. CCC=Cardiac conduction cells, CM=Cardiomyocytes, CT=Connective tissue. The arrows indicate myofibrils at the cell periphery.

In horses, CCCs were large and pale, and myofibrils were clear in the periphery of their cytoplasm (Figures 3a–d). The cardiomyocytes were dark, with a central nucleus and eosinophilic color (Figures 3a and b). In CCCs, we observed a larger area in the lateral region than in the posterior (p = 0.002; df = 3,65) or septal regions (p < 0.001; df = 3,65). This parameter was also higher in the anterior region than in the septal region (p = 0.013; df = 3,65). The maximum diameter of CCCs was greater in the lateral region than in the posterior (p = 0.001; df = 3,65) and septal (p < 0.001; df = 3,65) regions and was also significantly wider in the anterior region than in the septal region (p = 0.001; df = 3,65). Analyzing measurements of different morphometric parameters, we determined that CCCs were larger (55.74 ± 22.35 μm) than cardiomyocytes (24.38 ± 7.19 μm) in horses (Table 3). The diameter of CCCs in horses without cardiac disease (79.08 μm) was larger compared to cells (55.74 μm) in infarcted cases (p < 0.001; d = 0.5).

**Table 3:** Overall summary of morphometric parameters of cardiac conduction cells and cardiomyocytes of horses and dogs in MI.

Species	Cell	Area(μm^2^/SD)	Diameter maximum (μm/SD)	Diameter minimum (μm/SD)	Diameter mean (μm/SD)	Roundness (SD)
Horse	Cardiac conduction cell	1882.9 (1307.7)	55.74 (22.35)	37.08 (13.06)	45 (16.28)	1.21 (0.11)
Dog		463.9 (248.4)	26.83 (7.81)	19.46 (5.82)	22.90 (6.53)	1.13 (0.05)
Horse	Cardiomyocyte	407.6 (221)	24.38 (7.19)	18.41 (5.68)	21.19 (6.13)	1.09 (0.05)
Dog		273.3 (193.3)	19.91 (6)	14.86 (5.16)	17.28 (5.38)	1.09 (0.10)
	Diagram	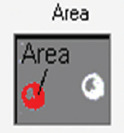	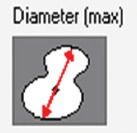	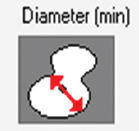	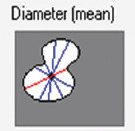	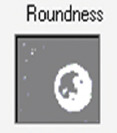

SD=Standard deviation, MI=Myocardial infarction

#### Dogs

**Figure 4:**
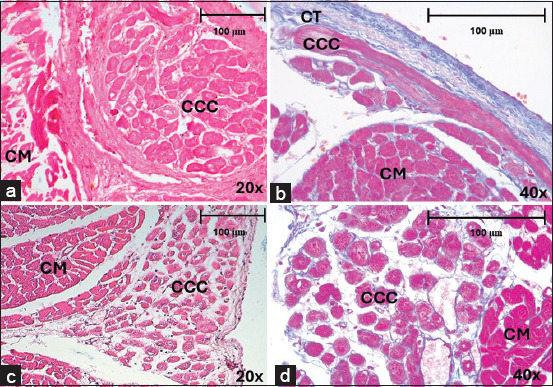
Morphology of cardiac conduction cells in the subendocardium of dogs in myocardial infarction, stained with (a–c) hematoxylin-eosin and (d) Masson’s trichrome. In the (a) posterior region at 20× and (b) anterior region at 40×. Septal region (c) at 20× and (d) 40×. CCC=Cardiac conduction cells, CM=Cardiomyocytes, CT=Connective tissue.

In dogs, CCCs were slightly paler than cardiomyocytes in some regions, while they were almost the same color in others and were difficult to recognize using hematoxylin-eosin (Figures 4a–c), although their identification improved using Masson’s trichrome (Figure 4d). These cells had a central nucleus, and no myofibrils were observed in the cytoplasm. The maximum diameter of CCCs was greater in the septal region than in the anterior region (p = 0.027; df = 3,86). These CCCs were slightly larger (26.83 ± 7.81 μm) than the surrounding cardiomyocytes (19.91 ± 6 μm) (Table 3). The area and diameters of the CCCs were greater in horses than in dogs (p < 0.001), whereas their roundness was greater in dogs than in horses (p < 0.001). The area and diameter of cardiomyocytes were larger in horses than in dogs (p < 0.001). In dogs, slight hypertrophy (31.21 μm) was observed in normal hearts when compared to hearts (26.83 μm) with MI (p = 0.114; d = 0.13).

**Figure 5:**
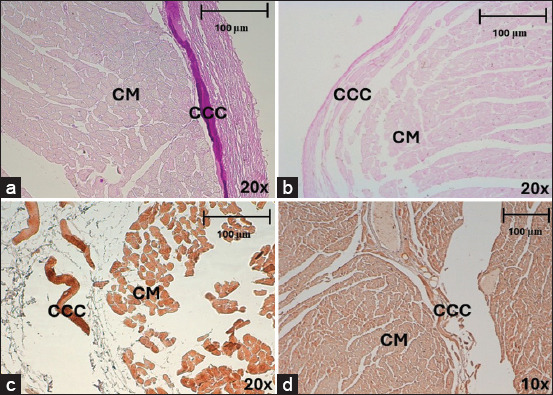
(a and b) CCCs detection using the PAS method in horses and dogs. (a) This method is used to identify intracytoplasmic glycogen in horses with myocardial infarction (MI), in the subendocardium, and in the septal region at 20×. (b) This method has little use in dogs with MI since it reveals negativity in the identification of intracytoplasmic glycogen in the conduction cells. (c and d) CCCs detection by immunohistochemical staining with desmin in horses and dogs. CCCs=Cardiac conduction cells, CM=Cardiomyocytes.

To improve CCC identification in infarct samples, we used a PAS method that was still useful in horses, revealing a large amount of glycogen accumulated in the cytoplasm of these cells (Figure 5a). In dogs, however, the method was not useful in heart attack cases, apparently due to the depletion of glycogen accumulated in the cytoplasm of the CCCs, which precluded their adequate identification (Figure 5b).

We also used immunohistochemical staining for desmin to enhance detection, finding these specific staining methods to be effective for CCC identification in horses (Figure 5c), but of little use in dogs, perhaps because the cytoplasmic content of these cells is affected when MI is present (Figure 5d).

### CFMJs

#### Horses

**Figure 6:**
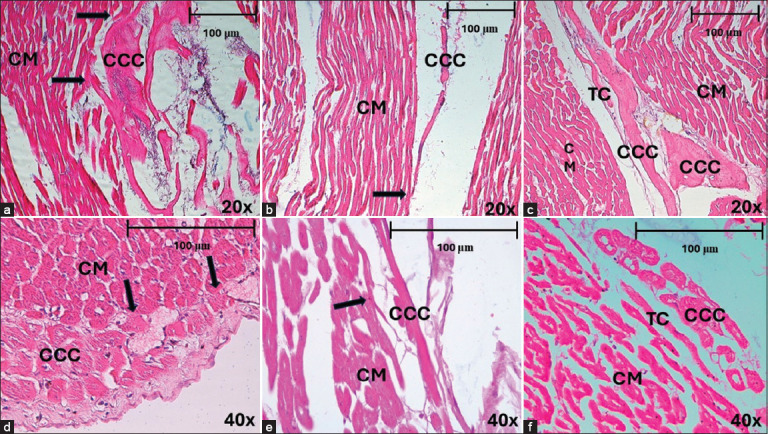
Different types of conduction fiber-myocardial junctions in horses and dogs after myocardial infarction, stained with hematoxylin-eosin (a) The arrows indicate a contact through cell bodies (CCB) junction in myocardium, in the septal region at 20× in horses. (b) The arrow reveals contact through cell prolongations (CCP) in myocardium in horses, in anterior region at 20×. (c) Note the contact through transitional cardiomyocytes (CTC) in myocardium in horses, in the anterior region at 20×. (d) Contact through cell bodies (CCB) junction in subendocardium, in anterior region at 40× in dogs. (e) Contact through cell prolongations (CCP) in subendocardium in dogs in the posterior region at 40×. (f) Contact through transitional cardiomyocytes (CTC) in subendocardium in dogsin the lateral region at 40×. CCC=Cardiac conduction cells, CM=Cardiomyocytes, TC=Transitional cells.

In horses, CFMJ was found in 36.7% of samples along the LV. We observed a higher percentage of CFMJ in the apex than in the other two slices (p < 0.001), since the junctions were mostly distributed toward the distal part of the heart. The CCB- and CCP-type junctions were found more frequently in the apex (Figures 6a and b) and the CTC-type junctions in the base (Figure 6c), a statistically significant finding (p < 0.001). CFMJs were found more frequently in the septal and lateral regions than in the other two regions (p < 0.001) (Table 4).

**Table 4:** Distribution of the junction type between cardiomyocytes and conduction cells in the slices, regions, and locations of the left ventricle.

Slice, region, and location	Junction type (%)					

	Horse			Dog		
	
	CCB	CCP	CTC	CCB	CCP	CTC
Slice						
Base	13.9	6.9	6.9	26.2	3.9	12.6
Middle third	27.6	0.3	5.3	27.6	1.8	4.1
Apex	45.4	1.5	5.8	25.5	4.3	5.3
Region						
Posterior	18.7	0.5	2.7	25.4	2.5	7.6
Septal	31.5	1.3	8.2	8.6	3.4	1.7
Anterior	23.7	1.7	5.2	35.5	2.7	10
Lateral	39.1	0.8	6.2	29.6	3.7	4.9
Location						
Subendocardium	19.3	1.3	8	29.4	1	0.4
Myocardium	40.2	0.9	4	-	-	-

CCB=Contact through cell bodies, CCP=Contact through cell prolongations, CTC=Contact through transitional cardiomyocytes

We observed greater CFMJ in the myocardium than in the subendocardium (p < 0.001). The CCB junction type was more frequently observed in the myocardium than in the CCP and CTC types in the subendocardium (p < 0.001) (Table 4).

#### Dogs

In dogs, 36.5% of the CFMJ cases were along the LV. The most frequent CCBs were observed in both slices and different regions. CFMJs were more frequently observed in the anterior region than other regions (p < 0.001). The CCB- and CTC-type junctions were more abundant in the anterior region (Figures 6d and f) and the CCP in the lateral region (p = 0.008) (Figure 6e) (Table 4).

### MI zone

#### Horses

**Figure 7:**
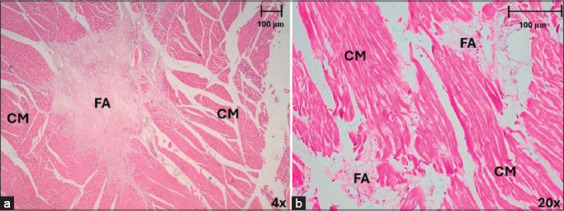
Area of myocardial infarction in horses and dogs stained with hematoxylin-eosin. (a) Large areas of fibrous scarring were observed in horses, mainly in the posterior region at 4×. (b) In dogs, small areas of fibrosis were recognized, distributed more frequently in the anterior and posterior regions at 20×. Note the lack of continuity of the muscle fibers due to the infarcted area and the presence of some inflammatory cells. FA=Fibrotic area, CM=Cardiomyocytes.

Cardiac muscle fiber lesions resulting from MI in horses were found mainly in the middle third, followed by the base and, to a lesser extent, in the apex. Regarding regions, myocardial lesions were equally distributed between the posterior, septal, and lateral regions, with no presence of lesions in the anterior region, although the most obvious damage was observed in the posterior region (Figure 7a). In the middle third and posterior region, we found extensive lesions, and in the other slices and regions, we observed moderate lesions and mild lesions in lesser quantities.

#### Dogs

In dogs, we observed only mild cardiac muscle lesions resulting from MI in the slices evaluated, but with a slight increase in the middle third. In terms of regions, we found these lesions posteriorly and less frequently in the anterior region (Figure 7b).

Mild lesions were observed, which were very small in size, with a minimal area of fibrous scarring and a slight loss of continuity of muscle fibers. Moderate lesions were found to be slightly larger, with coagulative necrosis of muscle fibers, little presence of inflammatory cells, and slight fibrous scar formation. In extensive lesions, we observed large areas of fibrous scarring, neovascularization, and moderate inflammatory cell presence.

## DISCUSSION

Understanding the conduction system in dogs and horses is crucial for understanding arrhythmic SD, a phenomenon with potentially fatal consequences. The intricate network of electrical pathways within the heart is essential for the regulation of heart rhythm and function. In both species, abnormalities in the conduction system can lead to arrhythmias, disrupting the heart’s ability to pump blood effectively. These arrhythmias may culminate in SD and pose significant challenges for owners, breeders, and veterinarians. By delving into the specifics of the conduction system in dogs and horses, including the anatomy, physiology, and common pathologies, veterinary professionals can better anticipate, diagnose, and manage arrhythmias, ultimately safeguarding the health and well-being of these beloved animals.

According to different studies in dogs, the most superficial CCFs were normal and showed no changes upon necrosis or infarction when this latter event was induced by occlusion of the coronary arteries, as in horses that died from SD [9, 17, 18]. Unlike previous study by Gómez-Torres *et al*. [1], our study showed altered CCF in each of the regions evaluated, based on comparing our study data with reports in animals under normal conditions in another previous study that we carried out using the same methodology. We observed that the density and thickness of CCFs in horses decreased to one-half or less of the normal values. Likewise, in dogs, the fiber density was reduced to half that of normal dogs, but the fiber thickness increased.

In dogs, Sharov *et al*. [18] have found glycogen depletion, signs of dissociation of the intercalated disc in CCCs, and even vacuolization of these cells. This decrease in glycogen content in the CCC of dogs was also observed in our study, with negative results obtained using the PAS method, possibly because of MI. This indicates that these cells may lose their capacity to resist ischemia and react faster than usual during heart attacks. PAS continues to be an effective identification method in horses, showing high positivity and CCC integrity in this animal species. We also observed that CCC size was smaller in infarcted horses (55.74 μm) than in normal hearts (77.40 μm) [1], demonstrating the damage caused by MI and the possible appearance of ventricular arrhythmias after the ischemic event. This has been corroborated by other authors who reported a decrease in CCC size with the mild presence of fibrosis upon MI [19]. In dogs, slight hypertrophy of these cells was observed compared to normal cases. In both species, cardiomyocytes presented mild hypertrophy, possibly due to pathological dilation arising from structural changes occurring in cells left without adequate oxygen after MI. Under normal conditions, desmin is used as a marker to differentiate CCCs from cardiomyocytes in both horses and dogs [22–25]. In our study, in MI, this method could still be used to differentiate CCCs in horses, but in dogs, poor positivity was observed, possibly due to a decrease in desmin filaments in the cell cytoplasm.

Under normal conditions, the presence of CFMJ in horses has been reported as 26.1% [1], lower than was found in our study where we observed these junctions in 36.7% of samples; this could be due to the need for greater transmission of the electrical impulse in the myocardium that is preserved after MI to try to preserve the contractile functionality of the cardiomyocytes. Echoing the study mentioned above, CFMJ was mainly observed in the apex, lateral region, and myocardium.

CFMJs have been observed in 30.8% of dogs under normal conditions [1]. In our study of infarcts, we observed these junctions in 36.5%, higher than in normal hearts, which, like in horses, could be due to the need to improve electrical transmission impulses after MI.

In studies in dogs in which MI was induced by occlusion of the coronary arteries, cardiomyocytes presented evident coagulative necrosis, eosinophilia, loss of striae and nuclei, and finally an inflammatory infiltrate with lymphocytes, neutrophils, and macrophages [9, 18]. In our study of dogs with MI, we found these same findings, but to a milder degree, with scarce loss of continuity of muscle fibers and inflammatory infiltrate.

Studies in horses that died from MI revealed different degrees of fibrosis, myocarditis, loss of continuity, and fatty infiltration of muscle fibers in the interventricular septum and right and left ventricular margins [1, 17, 19]. In our study, a slight modification was observed with the previously described histopathological pattern characterized by extensive areas of fibrosis, destruction of cardiac muscle fibers, and a large presence of inflammatory cells.

The occlusion processes over time in these species determining progressive alteration of the structures of the cardiac conduction system with clinical presence of arrhythmias merit preventive evaluations of veterinary cardiology to make early diagnoses and determine the required controls.

## CONCLUSION

This study provides a detailed histomorphometric analysis of CCFs and CCCs in horses and dogs following spontaneous MI. The findings reveal significant structural alterations in the conduction system, which may contribute to arrhythmogenesis and sudden cardiac death in these species. In horses, CCC diameter was significantly reduced in infarcted cases compared to normal hearts. In contrast, in dogs, slight hypertrophy was observed in normal hearts, but a reduction was noted post-MI. CCF density decreased in both species, while fiber thickness remained relatively unchanged. In horses, extensive myocardial fibrosis was observed, particularly in the middle third and posterior LV, whereas dogs exhibited mild, diffusely distributed lesions. The increased presence of CFMJs in both species post-MI suggests a compensatory response to maintain electrical conduction.

A major strength of this study lies in its rigorous histomorphometric and immunohistochemical approach, which enabled precise quantification of conduction fiber alterations and their correlation with myocardial fibrosis. Furthermore, by comparing infarcted hearts with previously studied normal specimens, this research provides novel insights into how conduction system structures respond to ischemic injury in veterinary cardiology.

However, some limitations should be acknowledged. The study sample was relatively small due to the inherent difficulty of obtaining postmortem hearts from infarcted animals. In addition, most of the specimens were from male animals, limiting the ability to assess potential sex-related differences in CCFs alterations. In some cases, the lack of prior clinical history also restricted the ability to correlate histological findings with pre-existing cardiac conditions.

Future research should focus on exploring the role of GAP junctions and their impact on electrical impulse transmission following MI and identifying molecular mechanisms underlying conduction disturbances. Further studies with a larger, more diverse sample size, including both sexes and animals with known clinical histories, would enhance the generalizability of these findings. In addition, translational research integrating pathophysiological findings with clinical management strategies could improve veterinary cardiology practices and advance therapeutic approaches for MI-related arrhythmias in companion and performance animals.

This study underscores the critical impact of MI on the structural integrity of the cardiac conduction system in horses and dogs, reinforcing the need for early diagnostic and preventive strategies to mitigate arrhythmogenic risks in these species.

## AUTHORS’ CONTRIBUTIONS

FGT, LBA, and ARS: Study conception and design, material preparation, and data collection and analysis. FGT: Drafted the manuscript and all authors have participated in the revision of the manuscript. All authors have read and approved the final manuscript.
